# Association between occupational ionizing radiation exposure duration and the increased risk of dyslipidemia: evidence from a large group of radiation workers

**DOI:** 10.3389/fpubh.2025.1651676

**Published:** 2025-08-18

**Authors:** Meimei Zhong, Xiaowen Wang, Qifeng Li, Zhijia Wu, Xiangyuan Huang, Xinyue Li, Yiqing Lian, Yingyi Peng, Zhiqiang Li, Zhifang Liu, Qia Wang, Yajun Gong, Peixia Hu, Xiaoyong Liu, Shuming Zhu, Shaomin Wu, Fangfang Zeng, Yiru Qin, Qiying Nong, Jinhan Wang, Yeqing Gu, Yongshun Huang, Wangjian Zhang, Na Zhao

**Affiliations:** ^1^Guangdong Province Hospital for Occupational Disease Prevention and Treatment, Guangzhou, China; ^2^School of Public Health, Shanxi Medical University, Taiyuan, China; ^3^Center for Public Health and Epidemic Preparedness and Response, School of Public Health, Peking University Health Science Center, Beijing, China; ^4^School of Public Health, Southern Medical University, Guangzhou, China; ^5^Department of Medical Statistics, School of Public Health, Sun Yat-sen University, Guangzhou, China; ^6^Tianjin Key Laboratory of Radiation Medicine and Molecular Nuclear Medicine, Institute of Radiation Medicine, Chinese Academy of Medical Sciences and Peking Union Medical College, Tianjin, China

**Keywords:** ionizing radiation, radiation worker, dyslipidemia, lipid biomarkers, mixed-effect model

## Abstract

**Background:**

Although radiation workers’ exposure levels consistently remained below established safety thresholds, accumulating evidence demonstrates that chronic low-dose ionizing radiation exposure may still pose significant health risks to humans. We aimed to explore the relationship between the years of low-dose radiation work and dyslipidemia.

**Methods:**

We collected occupational and physical examination data of 10,338 radiation workers from 1,200 workplaces during 2019–2020 in Guangdong Province, China. After controlling for social demographic and health behavior confounders, we used a mixed-effects model to assess the association of ionizing radiation exposure duration with blood lipid biomarkers as well as the prevalence of dyslipidemia. We further comprehensively evaluated the modifying effects of various demographic characteristics, health behavior factors, and air pollutant concentrations.

**Results:**

We found that participants with prolonged ionizing radiation exposure tended to have 8–40% higher levels of total cholesterol (TC) compared to those with < 10 years of exposure. The estimates were 9–23% for triglycerides (TG) and 5–26% for low-density lipoprotein cholesterol (LDL-C). Similar disparities were observed for the prevalence of overall dyslipidemia, abnormal TC or TG, hypercholesterolemia, hypertriglyceridemia, and high *β*-lipoproteinemia, with odds being 1.51–2.45 times higher in the group with > 30 years of ionizing radiation exposure compared to others. Our estimates further indicated greater effect estimates for prolonged ionizing radiation exposure and the prevalence of lipid abnormalities (*p* < 0.05) among the females, unmarried ones, and the workers with normal BMI.

**Conclusion:**

These findings suggest a deleterious effect of prolonged ionizing radiation exposure on lipid metabolism, with certain groups of workers being particularly vulnerable.

## 1 Introduction

Dyslipidemia is a common medical condition characterized by abnormal lipid profiles in the bloodstream. It is highly prevalent among Chinese adults (1), and has been responsible for the increased risk of a variety of cardiovascular diseases such as ischemic heart disease (IHD) and stroke (2). According to the estimates released by the WHO in 2012, dyslipidemia contributed to 18% of cases of IHD and 56% of strokes, resulting in over 4 million deaths globally each year (3).

It is widely recognized that chronic exposure to ionizing radiation increases the risk of cardiovascular diseases, likely mediated through the development of dyslipidemia. The association between ionizing radiation and disturbed lipid metabolism is biologically plausible. For example, Erica Werner et al. exposed human bronchial epithelial cells and lung cancer cell lines to ionizing radiation (5 Gy X radiation) and found a notable elevation in the activity of cholesterol biosynthesis enzymes and cellular cholesterol levels (10–30%) (4). Another study by Chukwuemeka N et al. exposed rats to 3 Gy of total body irradiation. After the initial exposure (5 days), serum triglyceride and total cholesterol levels increased significantly, with further increments observed after the second (10 days) and third (15 days) exposures (5). Changes in the level of cholesterol and triglycerides as observed in these experimental studies suggest the potential of ionizing radiation to disturb lipid metabolism (6). Moreover, emerging epidemiological evidence has shed light on the association between ionizing radiation exposure and dyslipidemia with its subclinical manifestations. Wen et al. recently demonstrated a significant correlation between chronic occupational radiation exposure and dyslipidemia among radiologists (7). Complementing these findings, Oslina et al. identified molecular alterations in blood samples of radiation-exposed workers that may indicate radiation-associated dyslipidemia linked to atherogenic progression (8).

Occupational exposure to ionizing radiation is believed to be higher than regular radiation from the environment among the general population (9). However, radiation workers are typically well-protected and exposed to minimal ionizing radiation, which remain significantly below the International Commission on Radiological Protection (ICRP)‘s safety threshold of an annual effective dose limit of 20 mSv/year, averaged over 5 years, with no single year exceeding 50 mSv for occupational exposure (10). Existing studies indicate that the vast majority of occupational radiation workers receive annual effective doses in the range of 1–5 mSv, well below the ICRP’s recommended limit (11–13). Although low-dose ionizing radiation generally would not lead to an apparent health impact in the short term, it is still unclear whether prolonged exposure to such low-dose ionizing radiation remains safe for humans. In situations where dose may not be a concern, the duration of such exposure may become a potential predictor for adverse health outcomes (14–17). However, existing research on the relationship between the duration of long-term low-dose exposure and blood lipid metabolism indicators remains extremely limited.

To address this important knowledge gap, we investigated a large group of radiation workers to clarify the relationship between their durations of ionizing radiation and the levels of multiple lipid biomarkers, as well as the prevalence of dyslipidemia.

## 2 Methods

### 2.1 Study design and population

**Figure 1:**
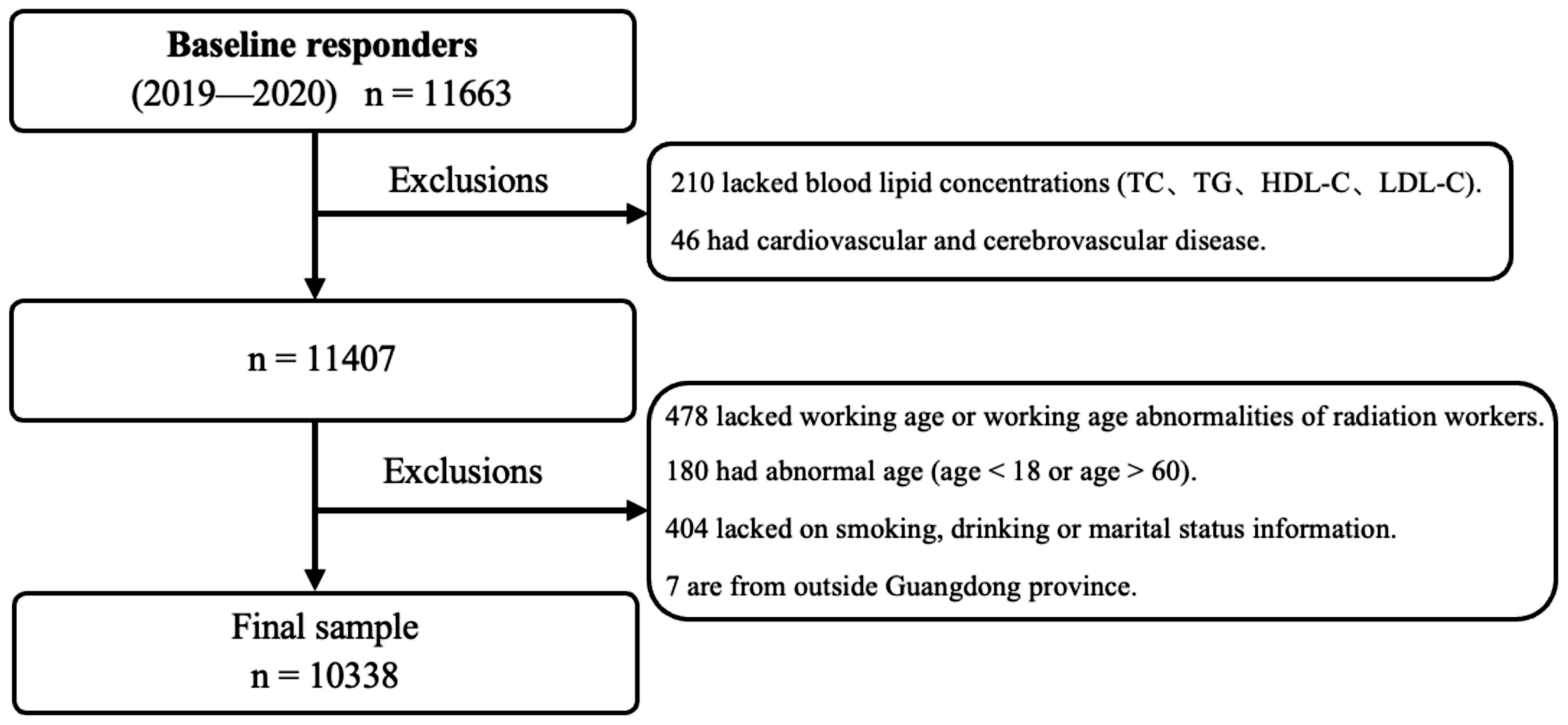
Cohort diagram illustrating the study population. The missing concentration of lipid index in the 210 participants was due to the lack of blood samples available for use in lipid analysis; The lack of information on smoking, drinking and marital status was due to incomplete information collection.

This study retrospectively reviewed health examination data from Guangdong Provincial Occupational Disease Prevention and Control Hospital, including 11,663 radiation workers from 1,200 institutions across the province between 2019 and 2020. Study participants were recruited from occupational sectors exposed to ionizing radiation, including medical radiology, nuclear industry operations, and industrial radiography. According to local regulations, radiation workers are required to participate in a health examination every 2 years. The study utilized retrospectively collected anonymized health examination records, thus requiring neither active recruitment nor direct interviews. Participant consent was waived under ethical approval (GDHOD MEC 2023034) in accordance with regulations governing research involving de-identified medical data. All the participants had at least 1 year of working experience and had no major health issues such as cancer or severe chronic conditions. Participants who 1. lacked information on lipid profiles, 2. had a family history of cardiovascular and cerebrovascular diseases, or 3. worked outside of Guangdong Province were excluded, resulting in a total of 10,338 participants in the analysis (Figure 1). The research protocol was approved by the Medical Ethics Committee of the Guangdong Provincial Hospital for Occupational Disease Prevention and Control (Approval No. GDHOD MEC 2023034).

### 2.2 Exposure measurement and covariates

Information on the duration of ionizing radiation exposure was collected from the individuals and confirmed by the employers. Occupational exposure duration was assessed using a dual-verification approach combining worker self-reported data with official employer records, thereby minimizing potential recall bias and ensuring data reliability. Participants were divided into four groups (in 10-year intervals): 1–10 years, 10–20 years, 20–30 years, and > 30 years. Before the physical examination, all participants underwent measurements of height and weight, and body mass index (BMI) was calculated from them. Information on basic demographic characteristics (i.e., age, gender, and marital status) and health behaviors (i.e., smoking and drinking) of the participants was obtained from the health examination database.

Since existing studies suggested a link between ambient air pollution and lipid indicators (18), we incorporated the concentration of major air pollutants, such as PM_2.5_, O_3_, SO_2_, and NO_2_ as potential confounders. The concentrations were determined for each participant by accessing the China High-resolution Air Pollution (CHAP) dataset according to the geocoded address of the workplace. The CHAP dataset is a well-validated, high-quality dataset on near-surface air pollution covering 10 km × 10 km spatially (19). It has been extensively utilized in previous research (20, 21). The average air pollution concentration over the year before the physical examination date was defined as the exposure variable. The distribution of pollutant concentrations (mean ± standard error) was as follows: 27.26 ± 2.66 μg/m^3^ for PM_2.5_, 103.35 ± 9.59 μg/m^3^ for O_3_, 8.58 ± 1.33 μg/m^3^ for SO_2_, and 41.76 ± 10.05 μg/m^3^ for NO_2_ (See Supplementary material).

### 2.3 Outcome definition

Blood samples were obtained following a fasting period of at least 10 h overnight. We used Mindray automatic analyzer (BS2000, China) to obtain the concentrations of lipid biomarkers, including TC, TG, high-density lipoprotein cholesterol (HDL-C), and LDL-C. Dyslipidemia was defined based on the established standards (22): 1. hypercholesterolemia (TC ≥ 6.2 mmol/L); 2. hypertriglyceridemia (TG ≥ 2.3 mmol/L); 3. hypolipoproteinemia (HDL-C < 1.0 mmol/L); 4. high *β* lipoproteinemia (LDL-C ≥ 4.1 mmol/L). The definition of overall dyslipidemia encompassed the occurrence of at least one of the four lipid abnormalities mentioned above.

### 2.4 Statistical analysis

We utilized mean (standard deviation, SD) for continuous variables, frequency (percentage) for categorical variables and using chi-square tests or analysis of variance to examine the disparities across different exposure duration groups, as appropriate. To assess the association between the duration of ionizing radiation exposure and lipid profiles, we developed three models using mixed-effects models.

Model 1. A crude model, only adjusted for a workplace-level random intercept.

Model 2. Further adjusted for age, gender, and marital status (i.e., married vs. unmarried/widowed/single) based on Model 1.

Model 3. Further adjusted for health behavior indicators, including BMI (kg/m^2^), smoking (i.e., non-smokers, light smokers with <100 cigarettes/year, moderate smokers with 100–200 cigarettes/year, heavy smokers with > 200 cigarettes/year), and drinking (i.e., yes or no), and additionally adjusted for the concentrations of air pollutants including PM_2.5_, SO_2_, O_3_, and NO_2_.

To evaluate the collinearity in the final model, we computed the variance inflation factor (VIF) to confirm that all variables in the model had VIF values < 5. Furthermore, we performed a comprehensive set of stratified analyses by baseline characteristics, health behaviors, and air pollution concentrations based on the final model. This allowed us to estimate how these factors modified the relationship between exposure duration and lipid levels, as well as dyslipidemia types.

To ensure the reliability of the findings, we carried out sensitivity analyses by (1) excluding the participants with an extremely short period of radiation working years (i.e., last 5%, *n* = 571) and (2) excluding those with hypertension (*n* = 1,344). Statistically significant estimates were identified with *p*-values below 0.05. All statistical analyses were performed utilizing R version 4.1.2.

## 3 Results

### 3.1 Participant characteristics

Among the 10,338 participants, 3,189 (30.8%) were female, and 8,121 (78.6%) were aged ≥ 30 years. The overall average BMI was 23.03 ± 3.39 kg/m^2^. Among the participants, 29.7% were overweight and 6.5% were obese. Additionally, we observed a significantly higher proportion of married ones than unmarried ones (76.8% vs. 23.2%). A total of 1,232 individuals (11.9%) were smokers, with 786 (7.6%) being classified as light smokers, 196 (1.9%) as moderate, and 250 (2.4%) as heavy smokers. A total of 1,949 individuals (18.9%) drank alcohol (Table 1). There were 2,671 individuals with dyslipidemia, among which 29.2% had 10–20 exposure years, 34.4% had 20–30 exposure years, and 41.8% had >30 exposure years. Compared to participants with normal blood lipids, those with dyslipidemia were likely to have more exposure years. Similar disparities were also observed with the criteria of abnormal blood lipids based on cholesterol, triglycerides, and LDL-C levels (Table 2).

**Table 1:** Characteristics according to radiation exposure years for study participants in 2019–2020.

Characteristics	Overall	Exposure years				*p*-values
		1–10	10–20	20–30	> 30	
Participants	10,338	5,427	2,619	1,574	718	
Exposure (ys)	11.68 (9.52)^a^	4.42 (2.45)	13.47 (2.88)	23.94 (2.93)	33.15 (2.93)	< 0.001
Gender						< 0.001
Male	7,149 (69.2)	3,567 (65.7)	1908 (72.9)	1,102 (70.0)	572 (79.7)	
Female	3,189 (30.8)	1860 (34.3)	711 (27.1)	472 (30.0)	146 (20.3)	
Age						< 0.001
< 30 years	2,247 (21.4)	2,205 (40.6)	12 (0.5)	0 (0.0)	0 (0.0)	
≥ 30 years	8,121 (78.6)	3,222 (59.4)	2,607 (99.5)	1,574 (100.0)	718 (100.0)	
Marriage						< 0.001
Married	7,944 (76.8)	3,290 (60.6)	2,424 (92.6)	1,527 (97.0)	703 (97.9)	
Unmarried/other	2,394 (23.2)	2,137 (39.4)	195 (7.4)	47 (3.0)	15 (2.1)	
Smoking						< 0.001
No-smokers	9,106 (88.1)	4,838 (89.1)	2,325 (88.8)	1,345 (85.5)	598 (83.3)	
Current smokers: mild	786 (7.6)	372 (6.9)	206 (7.9)	139 (8.8)	69 (9.6)	
Current smokers: moderate	196 (1.9)	99 (1.8)	39 (1.5)	40 (2.5)	18 (2.5)	
Current smokers: heavy	250 (2.4)	118 (2.2)	49 (1.9)	50 (3.2)	33 (4.6)	
Drinking						< 0.001
Yes	1949 (18.9)	895 (16.5)	557 (21.3)	332 (21.1)	165 (23.0)	
No	8,389 (81.1)	4,532 (83.5)	2062 (78.7)	1,242 (78.9)	553 (77.0)	
BMI						<0.001
< 24.0	6,595 (63.8)	3,803 (70.1)	1,546 (59.0)	859 (54.6)	387 (53.9)	
24.0 ~ 28.0	3,074 (29.7)	1,334 (24.6)	868 (33.1)	602 (38.2)	270 (37.6)	
> 28.0	669 (6.5)	290 (5.3)	205 (7.8)	113 (7.2)	61 (8.5)	
BMI (kg/m^2^)	23.03 (3.39)	22.50 (3.38)	23.51 (3.48)	23.65 (3.05)	23.95 (3.19)	< 0.001
Cholesterol (mmol/L)	5.09 (0.94)	4.92 (0.90)	5.14 (0.93)	5.39 (0.95)	5.51 (1.02)	< 0.001
Triglycerides (mmol/L)	1.42 (1.13)	1.27 (0.99)	1.51 (1.20)	1.65 (1.28)	1.72 (1.25)	< 0.001
HDL-C (mmol/L)	1.45 (0.36)	1.47 (0.36)	1.41 (0.35)	1.45 (0.38)	1.44 (0.39)	< 0.001
LDL-C (mmol/L)	2.99 (0.86)	2.87 (0.82)	3.04 (0.86)	3.19 (0.90)	3.29 (0.96)	< 0.001
Dyslipidemia^b^	2,671 (25.8)	1,063 (19.6)	766 (29.2)	542 (34.4)	300 (41.8)	< 0.001
Abnormal TC or TG	2,197 (21.3)	816 (15.0)	631 (24.1)	482 (30.6)	268 (37.3)	< 0.001
Abnormal TC	1,235 (11.9)	437 (8.1)	340 (13.0)	289 (18.4)	169 (23.5)	< 0.001
Abnormal TG	1,312 (12.7)	500 (9.2)	393 (15.0)	278 (17.7)	141 (19.6)	< 0.001
Abnormal HDL-C	760 (7.4)	343 (6.3)	234 (8.9)	123 (7.8)	60 (8.4)	< 0.001
Abnormal LDL-C	1,010 (9.8)	391 (7.2)	275 (10.5)	213 (13.5)	131 (18.2)	< 0.001

a, categorical variables were described as numbers (%) while continuous variables were described as mean (standard deviation). b, participants with at least one abnormal blood lipid profile.

**Table 2:** The effect of ionizing radiation exposure duration on dyslipidemia (categorical index).

Factors	Overall dyslipidemia	Abnormal TC or TG	Hypercholesterolemia	Hypercholesterolemia	Hypoalphalipoproteinemia	Hyperbetalipoproteinemia
	OR (95% CI)	OR (95% CI)	OR (95% CI)	OR (95% CI)	OR (95% CI)	OR (95% CI)
Model 1						
1–10 years	1 (ref)	1 (ref)	1 (ref)	1 (ref)	1 (ref)	1 (ref)
10–20 years	1.73 (1.55, 1.93)^a^	1.83 (1.62, 2.06)	1.71 (1.47, 1.99)	1.81 (1.56, 2.09)	1.51 (1.26, 1.80)	1.51 (1.28, 1.77)
20–30 years	2.28 (2.00, 2.59)	2.62 (2.28, 3.00)	2.57 (2.18, 3.02)	2.33 (1.97, 2.75)	1.36 (1.09, 1.69)	2.03 (1.70, 2.44)
> 30 years	3.09 (2.61, 3.66)	3.48 (2.92, 4.14)	3.39 (2.77, 4.15)	2.68 (2.16, 3.32)	1.51 (1.13, 2.03)	2.84 (2.27, 3.54)
p for trend	< 0.001	< 0.001	< 0.001	< 0.001	< 0.001	< 0.001
Model 2						
1–10 years	1 (ref)	1 (ref)	1 (ref)	1 (ref)	1 (ref)	1 (ref)
10–20 years	1.23 (1.09, 1.39)	1.31 (1.15, 1.49)	1.33 (1.13, 1.57)	1.26 (1.08, 1.48)	1.11 (0.92, 1.35)	1.16 (0.97, 1.39)
20–30 years	1.63 (1.42, 1.87)	1.88 (1.63, 2.18)	2.00 (1.68, 2.39)	1.62 (1.36, 1.94)	0.99 (0.79, 1.26)	1.58 (1.30, 1.92)
> 30 years	2.00 (1.68, 2.39)	2.30 (1.91, 2.77)	2.53 (2.04, 3.14)	1.65 (1.31, 2.06)	0.97 (0.71, 1.32)	2.07 (1.64, 2.62)
*p* for trend	< 0.001	< 0.001	< 0.001	< 0.001	0.883	< 0.001
Model 3						
1–10 years	1 (ref)	1 (ref)	1 (ref)	1 (ref)	1 (ref)	1 (ref)
10–20 years	1.19 (1.05, 1.35)	1.27 (1.11, 1.45)	1.31 (1.11, 1.54)	1.21 (1.03, 1.42)	1.05 (0.86, 1.28)	1.14 (0.96, 1.37)
20–30 years	1.52 (1.32, 1.75)	1.78 (1.53, 2.06)	1.94 (1.62, 2.32)	1.48 (1.23, 1.78)	0.87 (0.69, 1.11)	1.54 (1.26, 1.87)
> 30 years	1.90 (1.58, 2.27)	2.19 (1.81, 2.63)	2.45 (1.97, 3.04)	1.51 (1.20, 1.90)	0.86 (0.63, 1.18)	2.02 (1.60, 2.56)
*p* for trend	< 0.001	< 0.001	< 0.001	< 0.001	0.184	< 0.001

Overall dyslipidemia was defined as with at least one abnormal lipid profile; Abnormal TC or TG was defined as present either hypercholesterolemia or hypertriglyceridemia.

a, Effect estimates (i.e., odds ratio) with 95% confidence interval not covering 1 were considered.

### 3.2 Relationship of radiation exposure duration with blood lipid levels and dyslipidemia

We found that as exposure years increased, levels of TC, TG, and LDL-C increased in all models. After adjusting for covariates, the regression coefficient (*β*) slightly decreased but remained statistically significant (*p* < 0.05). However, radiation exposure had no significant influence on HDL-C levels. According to the results of the final model, compared to participants with 1–10 exposure years, those with >30 exposure years were observed with an increase of 0.40 (95% CI 0.33–0.48) mmol/L, 0.23 (95% CI 0.14–0.32) mmol/L and 0.26 (95% CI 0.19–0.33) mmol/L in serum concentrations of TC, TG, and LDL-C, respectively (Table 3).

**Table 3:** The effect of ionizing radiation exposure duration on lipid biomarkers (continuity index).

Factors	TC	TG	HDL-C	LDL-C
	Slope (95% CI)	Slope (95% CI)	Slope (95% CI)	Slope (95% CI)
Model 1				
1–10 years	0 (ref)	0 (ref)	0 (ref)	0 (ref)
10–20 years	0.20 (0.16, 0.25)^a^	0.26 (0.21, 0.31)	−0.07 (−0.09, −0.05)	0.16 (0.12, 0.20)
20–30 years	0.45 (0.40, 0.50)	0.42 (0.36, 0.48)	−0.04 (−0.06, −0.02)	0.31 (0.26, 0.36)
> 30 years	0.56 (0.49, 0.63)	0.50 (0.41, 0.58)	−0.06 (−0.09, −0.03)	0.40 (0.33, 0.46)
*p* for trend	< 0.001	< 0.001	< 0.001	< 0.001
Model 2				
1–10 years	0 (ref)	0 (ref)	0 (ref)	0 (ref)
10–20 years	0.09 (0.04, 0.13)	0.11 (0.05, 0.16)	−0.02 (−0.04, −0.00)	0.06 (0.02, 0.10)
20–30 years	0.34 (0.28, 0.39)	0.27 (0.20, 0.33)	0.01 (−0.01, 0.03)	0.21 (0.15, 0.26)
> 30 years	0.42 (0.34, 0.49)	0.28 (0.19, 0.36)	0.03 (0.01, 0.06)	0.27 (0.20, 0.33)
*p* for trend	< 0.001	< 0.001	0.057	< 0.001
Model 3				
1–10 years	0 (ref)	0 (ref)	0 (ref)	0 (ref)
10–20 years	0.08 (0.03, 0.13)	0.09 (0.03, 0.14)	−0.01 (−0.03, 0.00)	0.05 (0.01, 0.10)
20–30 years	0.32 (0.26, 0.38)	0.22 (0.15, 0.28)	0.03 (0.01, 0.05)	0.20 (0.15, 0.25)
> 30 years	0.40 (0.33, 0.48)	0.23 (0.14, 0 0.32)	0.04 (0.02, 0.07)	0.26 (0.19, 0.33)
*p* for trend	< 0.001	< 0.001	< 0.001	< 0.001

TC, total cholesterol; TG, triglycerides; HDL-C, high-density lipoprotein cholesterol; LDL-C, low-density lipoprotein cholesterol CI, confidence interval. a, Effect estimates (i.e., slope) with the 95% CI not covering 0 were considered statistically significant.

Consistent with the findings from continuous lipid spectrum observations, there was a positive exposure-response relationship between low-dose ionizing radiation exposure duration and the prevalence of dyslipidemia. Overall, the prevalence of dyslipidemia increased with increasing radiation exposure years. Compared to the group with the shortest exposure duration (1–10 years, the reference group), participants with prolonged radiation exposure tended to have a 73% (95% CI 1.55–1.93), 128% (95% CI 2.00–2.59), and 209% (95% CI 2.61–3.66) greater risk of dyslipidemia. After adjusting for important covariates, the odds ratios (95% CI) of dyslipidemia for groups with 10–20 years, 20–30 years, and > 30 years of exposure were 1.19 (95% CI 1.05–1.35), 1.52 (95% CI 1.32–1.75), and 1.90 (95% CI 1.58–2.27), respectively, relative to the reference group. We also found that the highest radiation exposure group (> 30 years) had significantly increased risks of overall dyslipidemia, abnormal TC or TG, hypercholesterolemia, hypertriglyceridemia, and high *β*-lipoproteinemia, with odds ratios of 1.90 (95% CI 1.58–2.27), 2.19 (95% CI 1.81–2.63), 2.45 (95% CI 1.97–3.04), 1.51 (95% CI 1.20–1.90), and 2.02 (95% CI 1.60–2.56), respectively. There was no significant association of radiation exposure years with the prevalence of hypo *α*-lipoproteinemia (*p* > 0.05) (Table 2).

### 3.3 Factors modifying the relationship between radiation exposure and blood lipid metabolism

We observed that the association of radiation exposure years with the prevalence of dyslipidemia significantly varied by gender (*p* for interaction < 0.001). Results in Tables 4, 5 suggested that female radiation workers were more susceptible to the effects of radiation exposure than males in terms of the levels of TC, LDL-C, and the prevalence of dyslipidemia. There was a significant interaction between marital status and radiation exposure years in the prevalence of dyslipidemia (*p* for interaction = 0.001). Unmarried/other participants in the high-exposure group (> 30 years) had a greater impact on the estimated effects (total cholesterol *β* = 0.95, 95% CI 0.51–1.39, *p* for interaction < 0.001; LDL-C *β* = 0.82, 95% CI 0.42–1.22, *p* for interaction = 0.006; overall dyslipidemia OR = 10.07, 95% CI 3.24–31.30, *p* for interaction = 0.001; abnormal TC or TG OR = 11.74, 95% CI 3.82–36.07, *p* for interaction < 0.001; hypercholesterolemia OR = 9.60, 95% CI 3.08–29.94, *p* for interaction = 0.007). A significant difference in the association between radiation exposure and overall dyslipidemia was found among BMI categories (*p* for interaction = 0.005). Obesity (BMI ≥ 28.0) and overweight (24.0 ≤ BMI < 28.0) appeared to be protective factors, with the lowest prevalence of overall dyslipidemia observed in obesity participants with low radiation exposure (10–20 years) (risk ratio 0.96, 95% CI 0.63–1.47). There was no significant interaction between smoking, drinking, and exposure years in the prevalence of dyslipidemia (*p* for interaction were 0.149 and 0.640, respectively). Within each smoking and drinking category, increasing radiation exposure duration was associated with an increased prevalence of overall dyslipidemia. Participants with 10–20 exposure years and smoking had the lowest risk (OR = 1.15, 95% CI 0.83–1.61). Non-drinkers in the 10–20 exposure years group had the lowest prevalence of dyslipidemia (OR = 1.16, 95% CI 1.01–1.33) (Tables 4, 5). Additionally, we observed that participants with long-term exposure to high concentrations of PM_2.5_, O_3_, and NO_2_ were more susceptible to the effects of radiation exposure years on overall dyslipidemia relative to their counterparts, although the interaction terms may not be statistically significant.

**Table 4:** The modification effect of basic participant characteristics on the association between ionizing radiation exposure duration and blood lipid profiles.

Factors	Total cholesterol (TC)		Triglyceride (TG)		HDL-C		LDL-C	
	Slope (95% CI)^a^	*p* ^b^	Slope (95% CI)	*p*	Slope (95% CI)	*p*	Slope (95% CI)	*p*
Gender		< 0.001		0.034		0.505		< 0.001
Male								
1–10 years	0 (ref)		0 (ref)		0 (ref)		0 (ref)	
10–20 years	0.08 (0.02, 0.14)		0.12 (0.04, 0.19)		−0.01 (−0.03, 0.00)		0.04 (−0.01, 0.10)	
20–30 years	0.30 (0.23, 0.37)		0.26 (0.17, 0.35)		0.02 (0.00, 0.05)		0.16 (0.09, 0.22)	
> 30 years	0.29 (0.21, 0.38)		0.24 (0.13, 0.35)		0.03 (0.01, 0.06)		0.15 (0.07, 0.24)	
Female								
1–10 years	0 (ref)		0 (ref)		0 (ref)		0 (ref)	
10–20 years	0.08 (0.00, 0.16)		0.01 (−0.05, 0.06)		−0.01 (−0.04, 0.03)		0.09 (0.01, 0.16)	
20–30 years	0.38 (0.29, 0.48)		0.13 (0.06, 0.19)		0.03 (−0.01, 0.07)		0.30 (0.21, 0.38)	
> 30 years	0.83 (0.68, 0.98)		0.21 (0.11, 0.31)		0.08 (0.02, 0.14)		0.65 (0.52, 0.79)	
Age		0.744		0.368		0.741		0.790
< 30 years								
1–10 years	0 (ref)		0 (ref)		0 (ref)		0 (ref)	
10–20 years	0.14 (−0.35, 0.62)		0.39 (−0.10, 0.88)		0.02 (−0.15, 0.20)		−0.06 (−0.50, 0.37)	
20–30 years	--		--		--		--	
> 30 years	--		--		--		--	
≥ 30 years								
1–10 years	0 (ref)		0 (ref)		0 (ref)		0 (ref)	
10–20 years	0.09 (0.04, 0.13)		0.09 (0.03, 0.14)		−0.01 (−0.03, 0.00)		0.06 (0.02, 0.11)	
20–30 years	0.33 (0.27, 0.39)		0.22 (0.15, 0.29)		0.03 (0.01, 0.04)		0.20 (0.15, 0.26)	
> 30 years	0.41 (0.33, 0.49)		0.23 (0.14, 0.32)		0.04 (0.02, 0.07)		0.27 (0.20, 0.34)	
Marriage		< 0.001		0.368		0.502		0.006
Married								
1–10 years	0 (ref)		0 (ref)		0 (ref)		0 (ref)	
10–20 years	0.06 (0.01, 0.11)		0.06 (0.00, 0.12)		−0.01 (−0.03, 0.01)		0.05 (0.00, 0.10)	
20–30 years	0.32 (0.26, 0.38)		0.21 (0.14, 0.28)		0.03 (0.01, 0.05)		0.20 (0.15, 0.26)	
> 30 years	0.39 (0.31, 0.46)		0.22 (0.12, 0.31)		0.04 (0.02, 0.07)		0.25 (0.18, 0.32)	
Unmarried/Other								
1–10 years	0 (ref)		0 (ref)		0 (ref)		0 (ref)	
10–20 years	0.29 (0.15, 0.43)		0.30 (0.14, 0.45)		−0.01 (−0.06, 0.04)		0.17 (0.04, 0.29)	
20–30 years	0.14 (−0.11, 0.40)		0.29 (0.01, 0.56)		−0.04 (−0.13, 0.05)		0.06 (0.17, 0.29)	
> 30 years	0.95 (0.51, 1.39)		0.26 (−0.22, 0.74)		0.04 (−0.11, 0.20)		0.82 (0.42, 1.22)	
Smoking		0.268		0.120		0.986		0.663
No								
1–10 years	0 (ref)		0 (ref)		0 (ref)		0 (ref)	
10–20 years	0.08 (0.03, 0.13)		0.08 (0.03, 0.14)		−0.01 (−0.03, 0.01)		0.05 (0.01, 0.10)	
20–30 years	0.34 (0.28, 0.40)		0.24 (0.18, 0.31)		0.03 (0.01, 0.05)		0.21 (0.15, 0.26)	
> 30 years	0.44 (0.36, 0.52)		0.24 (0.15, 0.33)		0.05 (0.02, 0.08)		0.28 (0.21, 0.36)	
Yes								
1–10 years	0 (ref)		0 (ref)		0 (ref)		0 (ref)	
10–20 years	0.09 (−0.06, 0.24)		0.08 (−0.15, 0.31)		−0.04 (−0.08, 0.01)		0.09 (−0.05, 0.23)	
20–30 years	0.18 (0.01, 0.35)		−0.01 (−0.26, 0.24)		0.02 (−0.03, 0.07)		0.16 (0.00, 0.32)	
> 30 years	0.22 (0.01, 0.43)		0.11 (−0.21, 0.42)		0.03 (−0.03, 0.09)		0.14 (−0.05, 0.34)	
Drinking		0.867		0.149		0.070		0.201
No								
1–10 years	0 (ref)		0 (ref)		0 (ref)		0 (ref)	
10–20 years	0.08 (0.03, 0.13)		0.06 (0.01, 0.12)		−0.01 (−0.02, 0.01)		0.06 (0.01, 0.10)	
20–30 years	0.33 (0.27, 0.39)		0.21 (0.15, 0.28)		0.02 (−0.00, 0.04)		0.22 (0.16, 0.27)	
> 30 years	0.42 (0.33, 0.50)		0.18 (0.09, 0.27)		0.05 (0.02, 0.08)		0.30 (0.22, 0.37)	
Yes								
1–10 years	0 (ref)		0 (ref)		0 (ref)		0 (ref)	
10–20 years	0.10 (−0.01, 0.22)		0.18 (0.03, 0.34)		−0.03 (−0.07, 0.00)		0.06 (−0.05, 0.16)	
20–30 years	0.29 (0.16, 0.42)		0.24 (0.05, 0.42)		0.05 (0.01, 0.09)		0.14 (0.02, 0.26)	
> 30 years	0.38 (0.22, 0.55)		0.40 (0.17, 0.63)		0.04 (−0.02, 0.09)		0.16 (0.01, 0.32)	
BMI		0.003		0.679		0.482		0.003
< 24.0								
1–10 years	0 (ref)		0 (ref)		0 (ref)		0 (ref)	
10–20 years	0.09 (0.03, 0.15)		0.07 (0.01, 0.13)		−0.02 (−0.04, 0.00)		0.08 (0.03, 0.13)	
20–30 years	0.37 (0.29, 0.44)		0.22 (0.14, 0.29)		0.02 (−0.00, 0.05)		0.25 (0.18, 0.31)	
> 30 years	0.44 (0.35, 0.54)		0.23 (0.13, 0.32)		0.04 (0.00, 0.08)		0.31 (0.22, 0.39)	
24.0 ~ 28.0								
1–10 years	0 (ref)		0 (ref)		0 (ref)		0 (ref)	
10–20 years	0.08 (−0.00, 0.17)		0.14 (0.02, 0.26)		−0.01 (−0.04, 0.02)		0.03 (−0.05, 0.12)	
20–30 years	0.31 (0.21, 0.40)		0.24 (0.10, 0.38)		0.02 (−0.01, 0.05)		0.18 (0.08, 0.27)	
> 30 years	0.41 (0.28, 0.54)		0.26 (0.08, 0.45)		0.06 (0.02, 0.10)		0.24 (0.12, 0.36)	
≥ 28.0								
1–10 years	0 (ref)		0 (ref)		0 (ref)		0 (ref)	
10–20 years	−0.02 (−0.21, 0.18)		−0.01 (−0.26, 0.24)		0.04 (−0.01, 0.09)		−0.05 (−0.23, 0.13)	
20–30 years	0.05 (−0.19, 0.28)		0.06 (−0.24, 0.37)		0.10 (0.04, 0.17)		−0.09 (−0.30, 0.13)	
> 30 years	0.14 (−0.15, 0.43)		0.04 (−0.33, 0.42)		0.08 (−0.00, 0.16)		0.04 (−0.23, 0.30)	
PM_2.5_^c^		0.291		0.792		0.097		0.570
Low PM_2.5_								
1–10 years	0 (ref)		0 (ref)		0 (ref)		0 (ref)	
10–20 years	0.04 (−0.03, 0.11)		0.05 (−0.03, 0.13)		−0.02 (−0.05, 0.00)		0.04 (−0.03, 0.10)	
20–30 years	0.26 (0.17, 0.35)		0.19 (0.09, 0.29)		0.02 (−0.01, 0.05)		0.15 (0.07, 0.23)	
> 30 years	0.39 (0.27, 0.50)		0.23 (0.10, 0.37)		0.01 (−0.03, 0.05)		0.26 (0.16, 0.37)	
High PM_2.5_								
1–10 years	0 (ref)		0 (ref)		0 (ref)		0 (ref)	
10–20 years	0.11 (0.04, 0.17)		0.11 (0.04, 0.19)		−0.00 (−0.03, 0.02)		0.06 (0.00, 0.12)	
20–30 years	0.37 (0.29, 0.44)		0.23 (0.14, 0.32)		0.03 (0.01, 0.06)		0.23 (0.16, 0.30)	
> 30 years	0.42 (0.32, 0.52)		0.22 (0.11, 0.33)		0.06 (0.03, 0.10)		0.26 (0.17, 0.35)	
O_3_		0.083		0.290		0.582		0.061
Low O_3_								
1–10 years	0 (ref)		0 (ref)		0 (ref)		0 (ref)	
10–20 years	0.08 (0.01, 0.15)		0.07 (−0.01, 0.15)		−0.00 (−0.03, 0.02)		0.05 (−0.01, 0.11)	
20–30 years	0.35 (0.27, 0.44)		0.16 (0.06, 0.26)		0.04 (0.01, 0.07)		0.24 (0.17, 0.32)	
> 30 years	0.30 (0.18, 0.42)		0.20 (0.06, 0.34)		0.05 (0.01, 0.09)		0.17 (0.06, 0.28)	
High O_3_								
1–10 years	0 (ref)		0 (ref)		0 (ref)		0 (ref)	
10–20 years	0.08 (0.01, 0.15)		0.10 (0.03, 0.18)		−0.02 (−0.05, 0.00)		0.06 (−0.00, 0.12)	
20–30 years	0.29 (0.22, 0.37)		0.26 (0.17, 0.34)		0.01 (−0.02, 0.04)		0.16 (0.09, 0.23)	
> 30 years	0.46 (0.36, 0.56)		0.24 (0.13, 0.35)		0.04 (0.00, 0.07)		0.31 (0.22, 0.40)	
SO_2_		0.212		0.350		0.373		0.182
Low SO_2_								
1–10 years	0 (ref)		0 (ref)		0 (ref)		0 (ref)	
10–20 years	0.10 (0.04, 0.16)		0.08 (0.01, 0.15)		−0.01 (−0.03, 0.01)		0.08 (0.02, 0.13)	
20–30 years	0.33 (0.26, 0.40)		0.22 (0.15, 0.30)		0.01 (−0.01, 0.04)		0.21 (0.15, 0.28)	
> 30 years	0.43 (0.34, 0.52)		0.23 (0.13, 0.33)		0.04 (0.01, 0.08)		0.29 (0.20, 0.37)	
High SO_2_								
1–10 years	0 (ref)		0 (ref)		0 (ref)		0 (ref)	
10–20 years	0.04 (−0.04, 0.12)		0.09 (−0.00, 0.19)		−0.02 (−0.04, 0.01)		0.02 (−0.06, 0.09)	
20–30 years	0.30 (0.21, 0.40)		0.18 (0.06, 0.30)		0.04 (0.01, 0.08)		0.18 (0.09, 0.27)	
> 30 years	0.33 (0.19, 0.47)		0.20 (0.03, 0.37)		0.03 (−0.02, 0.08)		0.21 (0.08, 0.34)	
NO_2_		0.568		0.849		0.694		0.331
Low NO_2_								
1–10 years	0 (ref)		0 (ref)		0 (ref)		0 (ref)	
10–20 years	0.08 (0.01, 0.15)		0.10 (0.02, 0.18)		−0.02 (−0.04, 0.00)		0.06 (−0.01, 0.12)	
20–30 years	0.28 (0.19, 0.36)		0.24 (0.14, 0.34)		0.03 (−0.00, 0.06)		0.14 (0.06, 0.22)	
> 30 years	0.38 (0.26, 0.51)		0.27 (0.12, 0.41)		0.04 (−0.01, 0.08)		0.23 (0.12, 0.34)	
High NO_2_								
1–10 years	0 (ref)		0 (ref)		0 (ref)		0 (ref)	
10–20 years	0.08 (0.01, 0.14)		0.07 (−0.00, 0.14)		−0.00 (−0.03, 0.02)		0.05 (−0.01, 0.11)	
20–30 years	0.35 (0.28, 0.43)		0.19 (0.11, 0.28)		0.03 (0.00, 0.05)		0.24 (0.17, 0.31)	
> 30 years	0.42 (0.32, 0.51)		0.20 (0.09, 0.31)		0.05 (0.02, 0.08)		0.28 (0.19, 0.37)	

a, Effect estimates with 95% confidence interval not covering 0 were considered statistically significant; b, *p* for interaction. c, The level of pollutants is defined according to the mean value of the pollutant concentration.

**Table 5:** The modification effect of basic participant characteristics on the association between ioniziang radiation exposure duration and dyslipidemia.

Factors	Overall dyslipidemia		Abnormal TC or TG		Hypercholesterolemia		Hypertriglyceridemia		Hypoalphalipoproteinemia		Hyperbetalipoproteinemia	
	OR (95% CI)^a^	*p* ^b^	OR (95% CI)	*p*	OR (95% CI)	*p*	OR (95% CI)	*p*	OR (95% CI)	*p*	OR (95% CI)	*p*
Gender		< 0.001		< 0.001		< 0.001		0.847		0.577		< 0.001
Male												
1–10 years	1 (ref)		1 (ref)		1 (ref)		1 (ref)		1 (ref)		1 (ref)	
10–20 years	1.18 (1.03, 1.35)		1.26 (1.09, 1.46)		1.21 (1.00, 1.46)		1.27 (1.07, 1.51)		1.04 (0.85, 1.27)		1.07 (0.88, 1.31)	
20–30 years	1.45 (1.23, 1.69)		1.71 (1.44, 2.02)		1.78 (1.45, 2.19)		1.55 (1.28, 1.89)		0.86 (0.67, 1.10)		1.42 (1.13, 1.77)	
> 30 years	1.62 (1.33, 1.97)		1.91 (1.56, 2.35)		1.96 (1.53, 2.51)		1.56 (1.23, 1.99)		0.86 (0.63, 1.18)		1.62 (1.24, 2.11)	
Female												
1–10 years	1 (ref)		1 (ref)		1 (ref)		1 (ref)		1 (ref)		1 (ref)	
10–20 years	1.20 (0.90, 1.60)		1.30 (0.96, 1.76)		1.66 (1.17, 2.36)		0.88 (0.53, 1.45)		1.41 (0.61, 3.30)		1.44 (0.96, 2.17)	
20–30 years	1.78 (1.32, 2.41)		2.06 (1.50, 2.81)		2.49 (1.73, 3.59)		1.11 (0.66, 1.88)		1.04 (0.38, 2.88)		2.05 (1.34, 3.15)	
> 30 years	3.94 (2.63, 5.88)		4.17 (2.76, 6.31)		5.73 (3.66, 8.97)		1.21 (0.54, 2.70)		0.87 (0.15, 4.93)		5.15 (3.10, 8.53)	
Age		0.571		1		0.987		0.712		0.102		0.839
< 30 years												
1–10 years	1 (ref)		1 (ref)		1 (ref)		1 (ref)		1 (ref)		1 (ref)	
10–20 years	0.61 (0.12, 3.16)		0.99 (0.20, 4.96)		1.12 (0.13, 9.98)		0.64 (0.08, 5.48)		0.00 (0.00, Inf)		1.18 (0.10, 13.42)	
20–30 years	--		--		--		--		--		--	
> 30 years	--		--		--		--		--		--	
≥ 30 years												
1–10 years	1 (ref)		1 (ref)		1 (ref)		1 (ref)		1 (ref)		1 (ref)	
10–20 years	1.22 (1.08, 1.38)		1.29 (1.13, 1.48)		1.33 (1.13, 1.57)		1.22 (1.04, 1.44)		1.08 (0.88, 1.31)		1.16 (0.97, 1.39)	
20–30 years	1.56 (1.35, 1.79)		1.81 (1.56, 2.10)		1.98 (1.65, 2.37)		1.50 (1.25, 1.80)		0.90 (0.71, 1.14)		1.56 (1.28, 1.90)	
> 30 years	1.94 (1.62, 2.32)		2.22 (1.84, 2.67)		2.49 (2.00, 3.10)		1.52 (1.21, 1.92)		0.90 (0.66, 1.22)		2.06 (1.63, 2.61)	
Marriage		0.001		< 0.001		0.007		0.143		0.238		0.072
Married												
1–10 years	1 (ref)		1 (ref)		1 (ref)		1 (ref)		1 (ref)		1 (ref)	
10–20 years	1.15 (1.01, 1.31)		1.22 (1.06, 1.40)		1.24 (1.04, 1.48)		1.17 (0.99, 1.39)		1.03 (0.84, 1.27)		1.11 (0.91, 1.34)	
20–30 years	1.53 (1.32, 1.77)		1.77 (1.52, 2.07)		1.93 (1.60, 2.32)		1.47 (1.22, 1.77)		0.87 (0.68, 1.12)		1.54 (1.26, 1.89)	
> 30 years	1.83 (1.52, 2.20)		2.09 (1.72, 2.52)		2.35 (1.88, 2.94)		1.46 (1.15, 1.84)		0.88 (0.64, 1.20)		1.97 (1.54, 2.51)	
Unmarried/Other												
1–10 years	1 (ref)		1 (ref)		1 (ref)		1 (ref)		1 (ref)		1 (ref)	
10–20 years	1.96 (1.32, 2.93)		2.13 (1.41, 3.22)		2.18 (1.34, 3.54)		1.69 (1.01, 2.84)		1.51 (0.79, 2.88)		1.85 (1.06, 3.21)	
20–30 years	1.23 (0.58, 2.63)		1.21 (0.55, 2.68)		1.51 (0.62, 3.68)		1.37 (0.54, 3.49)		1.29 (0.40, 4.19)		1.23 (0.44, 3.45)	
> 30 years	10.07 (3.24, 31.30)		11.74 (3.82, 36.07)		9.60 (3.08, 29.94)		5.19 (1.25, 21.53)		0.00 (0.00, Inf)		5.51 (1.51, 20.08)	
Smoking		0.149		0.231		0.481		0.500		0.673		0.919
No												
1–10 years	1 (ref)		1 (ref)		1 (ref)		1 (ref)		1 (ref)		1 (ref)	
10–20 years	1.20 (1.05, 1.37)		1.27 (1.10, 1.46)		1.31 (1.10, 1.57)		1.21 (1.01, 1.45)		1.02 (0.82, 1.27)		1.16 (0.75, 1.82)	
20–30 years	1.59 (1.36, 1.85)		1.87 (1.59, 2.20)		2.03 (1.68, 2.46)		1.56 (1.27, 1.91)		0.87 (0.66, 1.14)		0.84 (0.51, 1.40)	
> 30 years	2.02 (1.66, 2.46)		2.38 (1.95, 2.92)		2.65 (2.10, 3.35)		1.66 (1.29, 2.14)		0.85 (0.60, 1.22)		0.89 (0.47, 1.68)	
Yes												
1–10 years	1 (ref)		1 (ref)		1 (ref)		1 (ref)		1 (ref)		1 (ref)	
10–20 years	1.15 (0.83, 1.61)		1.24 (0.88, 1.75)		1.28 (0.81, 2.02)		1.13 (0.78, 1.64)		1.16 (0.75, 1.82)		1.43 (0.85, 2.39)	
20–30 years	1.17 (0.82, 1.69)		1.28 (0.88, 1.85)		1.50 (0.93, 2.42)		1.12 (0.75, 1.67)		0.84 (0.51, 1.40)		1.46 (0.84, 2.53)	
> 30 years	1.35 (0.86, 2.11)		1.38 (0.87, 2.18)		1.64 (0.93, 2.89)		1.00 (0.61, 1.66)		0.89 (0.47, 1.68)		2.07 (1.12, 3.83)	
Drinking		0.640		0.292		0.474		0.309		0.326		0.530
No												
1–10 years	1 (ref)		1 (ref)		1 (ref)		1 (ref)		1 (ref)		1 (ref)	
10–20 years	1.16 (1.01, 1.33)		1.22 (1.05, 1.41)		1.28 (1.06, 1.54)		1.12 (0.93, 1.35)		1.10 (0.88, 1.38)		1.09 (0.89, 1.33)	
20–30 years	1.51 (1.29, 1.77)		1.80 (1.52, 2.12)		2.04 (1.68, 2.49)		1.46 (1.19, 1.80)		0.91 (0.69, 1.20)		1.48 (1.19, 1.84)	
> 30 years	1.76 (1.43, 2.16)		2.01 (1.62, 2.49)		2.40 (1.88, 3.07)		1.31 (0.99, 1.72)		0.91 (0.63, 1.30)		1.88 (1.44, 2.46)	
Yes												
1–10 years	1 (ref)		1 (ref)		1 (ref)		1 (ref)		1 (ref)		1 (ref)	
10–20 years	1.32 (1.01, 1.72)		1.49 (1.12, 1.99)		1.44 (0.99, 2.08)		1.50 (1.08, 2.08)		0.90 (0.60, 1.33)		1.49 (0.98, 2.27)	
20–30 years	1.60 (1.18, 2.17)		1.73 (1.25, 2.40)		1.65 (1.09, 2.48)		1.56 (1.07, 2.27)		0.75 (0.46, 1.20)		1.89 (1.20, 2.98)	
> 30 years	2.48 (1.71, 3.61)		2.97 (2.01, 4.39)		2.72 (1.72, 4.31)		2.22 (1.43, 3.45)		0.70 (0.38, 1.30)		2.82 (1.70, 4.70)	
BMI		0.005		0.050		0.099		0.034		0.129		0.405
< 24.0												
1–10 years	1 (ref)		1 (ref)		1 (ref)		1 (ref)		1 (ref)		1 (ref)	
10–20 years	1.18 (1.00, 1.40)		1.20 (1.00, 1.44)		1.26 (1.01, 1.58)		1.14 (0.90, 1.46)		1.20 (0.88, 1.64)		1.14 (0.90, 1.46)	
20–30 years	1.70 (1.40, 2.07)		1.89 (1.54, 2.33)		2.13 (1.67, 2.70)		1.69 (1.29, 2.22)		1.06 (0.72, 1.56)		1.61 (1.23, 2.11)	
> 30 years	2.20 (1.72, 2.81)		2.33 (1.80, 3.02)		2.69 (2.01, 3.61)		1.73 (1.23, 2.42)		1.31 (0.82, 2.08)		2.30 (1.67, 3.17)	
24.0 ~ 28.0												
1–10 years	1 (ref)		1 (ref)		1 (ref)		1 (ref)		1 (ref)		1 (ref)	
10–20 years	1.25 (1.02, 1.53)		1.35 (1.09, 1.67)		1.43 (1.08, 1.89)		1.25 (0.98, 1.59)		0.95 (0.71, 1.27)		1.19 (0.89, 1.61)	
20–30 years	1.48 (1.18, 1.85)		1.78 (1.41, 2.25)		2.04 (1.51, 2.74)		1.37 (1.05, 1.79)		0.82 (0.59, 1.15)		1.64 (1.20, 2.25)	
>30 years	1.69 (1.26, 2.25)		2.15 (1.60, 2.88)		2.43 (1.70, 3.47)		1.47 (1.05, 2.07)		0.64 (0.40, 1.03)		1.75 (1.19, 2.58)	
≥ 28.0												
1–10 years	1 (ref)		1 (ref)		1 (ref)		1 (ref)		1 (ref)		1 (ref)	
10–20 years	0.96 (0.63, 1.47)		1.33 (0.85, 2.08)		1.10 (0.64, 1.88)		1.29 (0.79, 2.09)		0.96 (0.56, 1.64)		1.01 (0.56, 1.82)	
20–30 years	0.80 (0.48, 1.32)		1.11 (0.65, 1.89)		0.89 (0.46, 1.73)		1.10 (0.61, 1.99)		0.57 (0.28, 1.14)		0.89 (0.43, 1.82)	
> 30 years	1.30 (0.69, 2.43)		1.74 (0.91, 3.32)		1.82 (0.89, 3.72)		0.82 (0.39, 1.76)		0.65 (0.27, 1.53)		2.10 (1.00, 4.41)	
PM_2.5_^c^		0.054		0.109		0.146		0.629		0.881		0.276
Low PM_2.5_												
1–10 years	1 (ref)		1 (ref)		1 (ref)		1 (ref)		1 (ref)		1 (ref)	
10–20 years	1.06 (0.88, 1.27)		1.14 (0.93, 1.38)		1.09 (0.85, 1.40)		1.08 (0.86, 1.37)		1.03 (0.77, 1.37)		1.00 (0.77, 1.31)	
20–30 years	1.31 (1.06, 1.62)		1.54 (1.23, 1.93)		1.72 (1.31, 2.26)		1.30 (0.99, 1.71)		0.92 (0.65, 1.30)		1.38 (1.02, 1.86)	
> 30 years	1.73 (1.31, 2.29)		2.03 (1.52, 2.72)		2.28 (1.63, 3.19)		1.46 (1.03, 2.09)		0.76 (0.46, 1.25)		2.07 (1.45, 2.96)	
High PM_2.5_												
1–10 years	1 (ref)		1 (ref)		1 (ref)		1 (ref)		1 (ref)		1 (ref)	
10–20 years	1.32 (1.11, 1.56)		1.39 (1.16, 1.67)		1.51 (1.21, 1.88)		1.34 (1.07, 1.67)		1.08 (0.82, 1.41)		1.27 (1.00, 1.62)	
20–30 years	1.71 (1.41, 2.06)		1.95 (1.60, 2.38)		2.15 (1.69, 2.72)		1.61 (1.26, 2.06)		0.85 (0.61, 1.17)		1.68 (1.29, 2.18)	
> 30 years	2.04 (1.61, 2.59)		2.30 (1.80, 2.95)		2.63 (1.98, 3.50)		1.55 (1.14, 2.10)		0.95 (0.64, 1.41)		2.00 (1.46, 2.74)	
O_3_		0.230		0.306		0.041		0.429		0.528		0.022
Low O_3_												
1–10 years	1 (ref)		1 (ref)		1 (ref)		1 (ref)		1 (ref)		1 (ref)	
10–20 years	1.18 (0.99, 1.41)		1.22 (1.01, 1.48)		1.19 (0.93, 1.52)		1.25 (0.99, 1.57)		0.91 (0.69, 1.19)		1.26 (0.97, 1.64)	
20–30 years	1.63 (1.32, 2.01)		1.87 (1.50, 2.34)		2.28 (1.75, 2.98)		1.45 (1.10, 1.90)		0.83 (0.59, 1.16)		2.06 (1.54, 2.76)	
> 30 years	1.59 (1.19, 2.13)		1.91 (1.41, 2.58)		1.89 (1.31, 2.73)		1.49 (1.04, 2.14)		0.93 (0.60, 1.44)		1.61 (1.07, 2.42)	
High O_3_												
1–10 years	1 (ref)		1 (ref)		1 (ref)		1 (ref)		1 (ref)		1 (ref)	
10–20 years	1.21 (1.02, 1.44)		1.34 (1.11, 1.61)		1.41 (1.13, 1.77)		1.21 (0.96, 1.52)		1.25 (0.94, 1.67)		1.06 (0.83, 1.35)	
20–30 years	1.45 (1.20, 1.76)		1.72 (1.41, 2.11)		1.73 (1.36, 2.21)		1.52 (1.19, 1.95)		0.95 (0.67, 1.33)		1.21 (0.93, 1.58)	
> 30 years	2.11 (1.67, 2.66)		2.40 (1.89, 3.05)		2.82 (2.15, 3.72)		1.53 (1.13, 2.07)		0.82 (0.53, 1.27)		2.20 (1.64, 2.95)	
SO_2_		0.290		0.549		0.629		0.432		0.889		0.802
Low SO_2_												
1–10 years	1 (ref)		1 (ref)		1 (ref)		1 (ref)		1 (ref)		1 (ref)	
10–20 years	1.23 (1.06, 1.44)		1.31 (1.12, 1.55)		1.37 (1.12, 1.68)		1.20 (0.98, 1.47)		1.01 (0.78, 1.31)		1.18 (0.95, 1.48)	
20–30 years	1.63 (1.37, 1.94)		1.83 (1.53, 2.19)		1.91 (1.54, 2.38)		1.53 (1.22, 1.92)		0.87 (0.64, 1.18)		1.53 (1.20, 1.94)	
> 30 years	2.02 (1.63, 2.50)		2.30 (1.85, 2.86)		2.54 (1.97, 3.27)		1.57 (1.19, 2.06)		0.79 (0.53, 1.17)		2.07 (1.56, 2.73)	
High SO_2_												
1–10 years	1 (ref)		1 (ref)		1 (ref)		1 (ref)		1 (ref)		1 (ref)	
10–20 years	1.14 (0.93, 1.40)		1.22 (0.98, 1.53)		1.18 (0.89, 1.58)		1.27 (0.97, 1.67)		1.11 (0.82, 1.50)		1.08 (0.79, 1.46)	
20–30 years	1.34 (1.05, 1.72)		1.68 (1.29, 2.19)		1.99 (1.45, 2.73)		1.38 (1.00, 1.91)		0.89 (0.61, 1.30)		1.59 (1.13, 2.25)	
> 30 years	1.66 (1.18, 2.33)		1.96 (1.37, 2.81)		2.20 (1.44, 3.35)		1.38 (0.89, 2.14)		1.00 (0.60, 1.67)		1.90 (1.21, 2.96)	
NO_2_		0.336		0.317		0.144		0.933		0.527		0.289
Low NO_2_												
1–10 years	1 (ref)		1 (ref)		1 (ref)		1 (ref)		1 (ref)		1 (ref)	
10–20 years	1.07 (0.90, 1.28)		1.17 (0.97, 1.41)		1.09 (0.86, 1.40)		1.16 (0.93, 1.46)		1.03 (0.79, 1.34)		1.03 (0.79, 1.33)	
20–30 years	1.30 (1.05, 1.60)		1.55 (1.24, 1.93)		1.55 (1.17, 2.04)		1.45 (1.12, 1.88)		0.84 (0.60, 1.18)		1.22 (0.90, 1.65)	
> 30 years	1.73 (1.29, 2.31)		2.07 (1.53, 2.80)		2.05 (1.43, 2.93)		1.57 (1.11, 2.23)		0.70 (0.42, 1.16)		1.78 (1.21, 2.60)	
High NO_2_												
1–10 years	1 (ref)		1 (ref)		1 (ref)		1 (ref)		1 (ref)		1 (ref)	
10–20 years	1.32 (1.11, 1.57)		1.36 (1.13, 1.64)		1.53 (1.22, 1.92)		1.24 (0.99, 1.57)		1.09 (0.81, 1.45)		1.29 (1.00, 1.65)	
20–30 years	1.73 (1.43, 2.10)		1.94 (1.59, 2.37)		2.30 (1.81, 2.93)		1.46 (1.13, 1.89)		0.93 (0.67, 1.30)		1.85 (1.42, 2.41)	
> 30 years	2.07 (1.64, 2.62)		2.28 (1.79, 2.90)		2.87 (2.17, 3.79)		1.45 (1.07, 1.97)		1.02 (0.68, 1.53)		2.29 (1.69, 3.11)	

a, Effect estimates with 95% CI not covering 0 were considered statistically significant; b, *p* for interaction; c, The level of pollutants is defined according to the mean value of the pollutant concentration.

We observed similar effect estimates when excluding participants with a working experience of 1 year or less or excluding those with hypertension (See Supplementary material).

## 4 Discussion

Our results confirmed that occupational exposure duration to ionizing radiation was significantly associated with higher levels of TC, TG, and LDL-C, as well as a higher risk of blood lipid abnormalities. We found the association differed by gender and marriage, with females and those unmarried being more susceptible. Participants with obesity (≥ 28.0 kg/m^2^) tended to have a lower risk of blood lipid abnormalities following exposure.

### 4.1 Interpretation of the results

Recent evidence has consistently demonstrated the potential link between ionizing radiation exposure and dyslipidemias. In a rat model, ionizing radiation exposure altered lipid metabolism, resulting in significant increases in TC, TG, and LDL-C, as well as decreases in HDL-C concentration. Furthermore, Amorim et al. and Daniel et al. found elevated levels of triglycerides and LDL-C, and free fatty acids decades after radiation (23, 24), indicating impaired lipid metabolism related to the radiation exposure (25). An increased risk of dyslipidemia and coronary heart disease was also observed after radiation therapy (26). In epidemiological studies, Wen et al. observed a dose-dependent increase in dyslipidemia risk and elevated TG levels, with the systemic immune-inflammation index (SII) potentially mediating the association between cumulative radiation dose and TG concentrations (7). Research by Oslina et al. revealed blood molecular alterations in individuals with occupational exposure to dual radiation sources (*α*-particles internally and *γ*-rays externally), potentially marking dyslipidemia connected to atherogenic processes (8). Although the link between ionizing radiation exposure and dyslipidemias has been investigated, the epidemiological evidence is still limited (7, 8). Therefore, our study significantly contributes to the epidemiological evidence regarding the association between ionizing radiation exposure and dyslipidemias. It is noteworthy that the OR of dyslipidemia for the groups with 20–30 exposure years and > 30 exposure years could increase by 52% (95% CI 1.32–1.75) and 90% (95% CI 1.58–2.27), relative to the reference group.

Our study suggested multiple effect modifiers underlying the association between ionizing radiation exposure and serum lipid profile, including gender, marital status and BMI. Specifically, we observed greater effect estimates among the females, the unmarried, and the workers with normal BMI. Although existing evidence is limited on the human vulnerability toward the association between ionizing radiation and dyslipidemia, some studies suggested that these subgroups were more susceptible to the impact of ionizing radiation on other outcomes. Similar interpretations may help understanding the observed phenomenon of this study. For example, a study reviewed the primary human studies concerning the health risks associated with radiation exposure, revealing that females exposed to the same dose of radiation may face a significantly higher risk of experiencing and succumbing to radiation-induced cancer compared to males (27). Sex leads to genetic differences, hormonal regulation and DNA damage, which are considered possible driving mechanisms of radiation sensitivity and radiation-induced risk of dyslipidemia (28). Furthermore, a cohort study of industrial radiographers in Xinjiang, China, found that the long-term low-dose radiation exposure posed a risk for liver injury in the unadjusted analysis, with unmarried workers at increased risk compared to married workers. The mechanisms behind this may involve differences in lifestyle, hormonal roles, stress levels, or access to healthcare between married and single individuals, which interact with radiation exposure (29). Furthermore, the existing research on the impact of BMI on worker radiation exposure estimates is inconsistent. A cross-sectional study on the association between BMI and abdominal fat in preoperative liver CT scans of potential live donor liver and radiation dose showed that obese patients typically receive higher effective doses of ionizing radiation in imaging examinations compared to normal weight individuals, as the thickness of the surveyed area in obese individuals is larger, leading to higher radiation exposure (30, 31). The discrepancy between these findings and the outcomes of our study may be partly attributable to our use of BMI as a practical indicator of general obesity, derived from routinely collected occupational health records. However, abdominal obesity, particularly visceral fat, has been shown to play a more significant role in lipid metabolism dysregulation than BMI alone (32–34). More studies are needed in the future to replicate these findings and clarify the underlying causes.

### 4.2 Biological rationality

Ionizing radiation (IR) is commonly employed in both diagnostic and therapeutic applications. However, it is important to note that healthcare workers may inadvertently be exposed to IR (35). Although the levels of radiation experienced by healthcare workers are typically low, the cumulative ionizing dose over several years of work may have adverse effects on human health (13).

Several potential mechanisms have been suggested for the link between exposure to ionizing radiation and changes in the lipid spectrum. Ionizing radiation could induce excessive lipid peroxidation and reduce antioxidant levels, leading to oxidative stress and thereby disrupting lipid metabolism. This is also a key mechanism for regulating a programmed form of cell death in ferroptosis (36). The second mechanism is that ionizing radiation may disrupt the feedback regulation of cholesterol biosynthesis, leading to elevated levels of cholesterol in lung and possibly other possible post-mitotic tissues (4). This could affect the function and cellular processes of cholesterol-sensitive proteins. Furthermore, ionizing radiation can directly interact with and damage cell membranes, leading to lipid peroxidation and subsequent apoptosis and necrosis (37). Furthermore, radiation exposure could cause skin lipid remodeling, including changes in fatty acid and lipid composition in the skin, which may lead to radiation-induced skin lipid damage (38). Another possible mechanism is that radiation-induced increases in mitochondrial damage and insulin resistance could also dysregulate the lipid metabolism and insulin resistance profiles (39). Moreover, the epigenetic changes induced by IR may contribute to the changes in the expression of genes involved in lipid metabolism (40). Together, the existing evidence suggests that the deleterious impact of prolonged radiation exposure on lipid metabolism we observed is biologically plausible.

### 4.3 Strengths and limitations

Population-based epidemiological evidence demonstrated significant associations between chronic low-dose ionizing radiation exposure and dyslipidemia indicators, providing crucial scientific support for health risk evaluation in long-term radiation exposure settings. The study covered participants from 1,200 workplaces in Guangdong Province which enhances the applicability of our studies. The extensive sample size ensures ample statistical power to test moderate correlations and estimate variations in those association among various subgroups. Additionally, we established various lipid variables to comprehensively investigate the effect of radiation on lipid metabolism. Our findings have been validated through multiple sensitivity analyses.

However, some limitations should be acknowledged. First, we used occupational exposure duration to represent the ionizing radiation exposure since the individual-level radiation dose tended to be low and remains unknown. While duration of employment serves as a critical exposure metric under chronic low-dose conditions, individual dose data are also essential. However, due to limitations in data accessibility, this study did not incorporate individual dose measurements. Future studies should prioritize the collection of individual dose data to refine exposure assessments. Obtaining individual exposure data through dosimeters is feasible, as radiation workers could be required to wear personal monitoring devices. However, challenges include ensuring consistent device use, standardizing data collection across workplaces, and integrating historical dose records. Future studies should prioritize collaboration with occupational health agencies to access these data, enabling more precise dose–response analyses (41). Second, as this is a cross-sectional study, we could not establish the causal link between ionizing radiation exposure and lipid metabolism. The cross-sectional study design is susceptible to numerous confounding factors that can bias results. For instance, background prevalence, which varies by geographical location, age, or socioeconomic status of the study population, may skew findings. Other confounders include selection bias (e.g., underrepresentation in the sample), information bias (e.g., recall bias or measurement errors), temporal confounding (e.g., difficulty distinguishing the sequence of exposure and outcome), and genetic factors that may simultaneously influence the exposure-outcome relationship. These issues can compromise the reliability of causal inferences. Future research may require more rigorous designs with stronger causal inference capabilities, such as cohort studies. Third, we did not collect individual-level data on dietary habits and lifestyle factors, including physical activity, diet and exercise levels. These factors are known to influence lipid metabolism and may also modulate biological responses to ionizing radiation. The lack of such data introduces the possibility of residual confounding that may affect the interpretation of our results. Due to the retrospective design of the study, it was not feasible to collect this information. Future investigations should incorporate validated tools such as food frequency questionnaires, physical activity surveys, or relevant biomarkers to comprehensively assess lifestyle and behavioral factors. These approaches will help to reduce residual confounding and enhance the validity and generalizability of the findings.

## 5 Conclusion

Our study suggests that radiation workers with longer-term occupational ionizing radiation exposure have a higher risk of disturbed lipid levels. Females, unmarried individuals, and workers with normal BMI, appear to be more vulnerable. Further studies are still warranted to validate our results. Further validation in other radiation worker cohorts is needed to confirm its occupational health implications.

## Data Availability

The raw data supporting the conclusions of this article will be made available by the authors, without undue reservation.
