# Supplementary material for: Association between occupational ionizing radiation exposure duration and the increased risk of dyslipidemia: evidence from a large group of radiation workers
Source: Front Public Health. 2025 Aug 18;13:1651676. doi: 10.3389/fpubh.2025.1651676 (PMC12401101; doi:10.3389/fpubh.2025.1651676)
Supplement: Supplementary file 1 [file Table_1.docx]

**Table S1. 1-Year average concentrations of ambient air pollutants.**

| **Pollutants** | **Subjects *n*** | ***Mean* ± *SD* μg/m^3^** | ***Min* μg/m^3^** | ***Max* μg/m^3^** | ***IQR* μg/m^3^** | **Pearson correlation coefficients** | | | | |
| --- | --- | --- | --- | --- | --- | --- | --- | --- | --- | --- |
|  |  |  |  |  |  | **PM_2.5_** | **PM_10_** | **O_3_** | **SO_2_** | **NO_2_** |
| **PM_2.5_** | 10390 | 27.25±2.65 | 19.12 | 34.47 | 3.44 | 1 | 0.83 | -0.19 | 0.42 | 0.53 |
| **PM_10_** | 10390 | 47.73±3.85 | 33.00 | 59.48 | 4.90 |  | 1 | 0.19 | 0.19 | 0.67 |
| **O_3_** | 10390 | 103.40±9.58 | 73.27 | 123.35 | 17.38 |  |  | 1 | -0.53 | 0.19 |
| **SO_2_** | 10390 | 8.57±1.33 | 5.65 | 15.59 | 1.379 |  |  |  | 1 | -0.11 |
| **NO_2_** | 10390 | 41.65±10.05 | 11.32 | 60.95 | 12.95 |  |  |  |  | 1 |

*SD*: standard error.

*Min*: minimum value.

*Max*: maximum value.

**Table S2. The effect of ionizing radiation exposure duration on dyslipidemia (continuity index).**

| **Factors** | **TC** | **TG** | **HDL-C** | **LDL-C** |
| --- | --- | --- | --- | --- |
|  | ***Slope* (95% *CI*）** | ***Slope*（95% *CI*）** | ***Slope* (95% *CI*)** | **Slo*pe* (95% *CI*)** |
| **Model 1** |  |  |  |  |
| 1-10 years | 0 (ref) | 0 (ref) | 0 (ref) | 0 (ref) |
| 10-20 years | 0.19 (0.15, 0.24)^a^ | 0.25 (0.20, 0.30) | -0.07 (-0.09, -0.05) | 0.15 (0.11, 0.19) |
| 20-30 years | 0.44 (0.39, 0.50) | 0.41 (0.35, 0.48) | -0.04 (-0.06, -0.02) | 0.30 (0.25, 0.35) |
| > 30 years | 0.55 (0.47, 0.62) | 0.49 (0.40, 0.58) | -0.06 (-0.09, -0.03) | 0.39 (0.32, 0.45) |
| *P* for trend | < 0.001 | < 0.001 | < 0.001 | < 0.001 |
| **Model 2** |  |  |  |  |
| 1-10 years | 0 (ref) | 0 (ref) | 0 (ref) | 0 (ref) |
| 10-20 years | 0.09 (0.05, 0.14) | 0.11 (0.05, 0.16) | -0.02 (-0.04, -0.00) | 0.07 (0.02, 0.11) |
| 20-30 years | 0.34 (0.29, 0.40) | 0.27 (0.20, 0.33) | 0.01 (-0.01, 0.03) | 0.21 (0.16, 0.27) |
| > 30 years | 0.43 (0.35, 0.50) | 0.27 (0.19, 0.36) | 0.03 (0.00, 0.06) | 0.27 (0.20, 0.34) |
| *P* for trend | < 0.001 | < 0.001 | 0.057 | < 0.001 |
| **Model 3** |  |  |  |  |
| 1-10 years | 0 (ref) | 0 (ref) | 0 (ref) | 0 (ref) |
| 10-20 years | 0.09 (0.04, 0.14) | 0.09 (0.03, 0.14) | -0.01 (-0.03, 0.00) | 0.06 (0.02, 0.11) |
| 20-30 years | 0.33 (0.27, 0.39) | 0.22 (0.15, 0.29) | 0.02 (0.01, 0.04) | 0.21 (0.15, 0.26) |
| > 30 years | 0.41 (0.34, 0.49) | 0.24 (0.15, 0.33) | 0.04 (0.01, 0.07) | 0.27 (0.20, 0.34) |
| *P* for trend | < 0.001 | < 0.001 | < 0.001 | < 0.001 |

a: Effect estimates (i.e., slope) with the 95% *CI* not covering 0 were considered statistically significant.

**Table S3. The effect of ionizing radiation exposure duration on dyslipidemia (categorical index).**

| **Factors** | **Overall dyslipidemia^a^** | **Abnormal TC or TG** | **Hypercholesterolemia** | **Hypertriglyceridemia** | **Hypoalphalipoproteinemia** | **Hyperbetalipoproteinemia** |
| --- | --- | --- | --- | --- | --- | --- |
|  | ***OR* (95% *CI*)** | ***OR* (95% *CI*)** | ***OR* (95% *CI*)** | ***OR* (95% *CI*)** | ***OR* (95% *CI*)** | ***OR* (95% *CI*)** |
| **Model 1** |  |  |  |  |  |  |
| 1-10 years | 1 (ref) | 1 (ref) | 1 (ref) | 1 (ref) | 1 (ref) | 1 (ref) |
| 10-20 years | 1.71 (1.53, 1.92)^b^ | 1.80 (1.59, 2.03) | 1.68 (1.44, 1.96) | 1.78 (1.53, 2.06) | 1.50 (1.25, 1.79) | 1.49 (1.26, 1.76) |
| 20-30 years | 2.25 (1.97, 2.56) | 2.57 (2.24, 2.96) | 2.52 (2.13, 2.98) | 2.29 (1.93, 2.71) | 1.35 (1.08, 1.69) | 2.00 (1.67, 2.41) |
| > 30 years | 3.05 (2.57, 3.61) | 3.41 (2.86, 4.07) | 3.32 (2.71, 4.08) | 2.63 (2.11, 3.26) | 1.51 (1.12, 2.03) | 2.79 (2.23, 3.49) |
| *P* for trend | < 0.001 | < 0.001 | < 0.001 | < 0.001 | < 0.001 | <0.001 |
| **Model 2** |  |  |  |  |  |  |
| 1-10 years | 1 (ref) | 1 (ref) | 1 (ref) | 1 (ref) | 1 (ref) | 1 (ref) |
| 10-20 years | 1.26 (1.11, 1.42) | 1.33 (1.16, 1.51) | 1.36 (1.15, 1.61) | 1.26 (1.07, 1.48) | 1.13 (0.92, 1.37) | 1.21 (1.01, 1.46) |
| 20-30 years | 1.66 (1.44, 1.91) | 1.91 (1.65, 2.22) | 2.05 (1.71, 2.46) | 1.63 (1.36, 1.95) | 1.01 (0.80, 1.28) | 1.65 (1.35, 2.01) |
| > 30 years | 2.05 (1.71, 2.45) | 2.34 (1.94, 2.81) | 2.60 (2.09, 3.23) | 1.65 (1.31, 2.07) | 0.98 (0.72, 1.34) | 2.16 (1.70, 2.74) |
| *P* for trend | < 0.001 | < 0.001 | < 0.001 | < 0.001 | 0.824 | <0.001 |
| **Model 3** |  |  |  |  |  |  |
| 1-10 years | 1 (ref) | 1 (ref) | 1 (ref) | 1 (ref) | 1 (ref) | 1 (ref) |
| 10-20 years | 1.22 (1.08, 1.38) | 1.29 (1.13, 1.48) | 1.34 (1.13, 1.58) | 1.22 (1.03, 1.43) | 1.08 (0.88, 1.31) | 1.19 (0.99, 1.43) |
| 20-30 years | 1.56 (1.35, 1.80) | 1.81 (1.56, 2.10) | 1.99 (1.66, 2.38) | 1.49 (1.24, 1.79) | 0.90 (0.70, 1.14) | 1.60 (1.31, 1.96) |
| > 30 years | 1.98 (1.65, 2.38) | 2.26 (1.87, 2.72) | 2.53 (2.03, 3.15) | 1.55 (1.23, 1.95) | 0.91 (0.67, 1.24) | 2.13 (1.67, 2.70) |
| *P* for trend | < 0.001 | < 0.001 | < 0.001 | < 0.001 | 0.312 | <0.001 |

a: Overall dyslipidemia was defined as with at least one abnormal lipid profile.

Abnormal TC or TG: was defined as present either hypercholesterolemia or hypertriglyceridemia, they are the two most common dyslipidemia conditions.

b: Effect estimates (i.e., odds ratio) with 95% *CI* not covering 1 were considered.

**Table S4. The effect of ionizing radiation exposure duration on dyslipidemia (continuity index).**

| **Factors** | **TC** | **TG** | **HDL-C** | **LDL-C** |
| --- | --- | --- | --- | --- |
|  | ***Slope* (95% *CI*)** | ***Slope* (95% *CI*)** | ***Slope* (95% *CI*)** | ***Slope* (95% *CI*)** |
| **Model 1** |  |  |  |  |
| 1-10 years | 0 (ref) | 0 (ref) | 0 (ref) | 0 (ref) |
| 10-20 years | 0.19 (0.15, 0.24)^a^ | 0.25 (0.20, 0.30) | -0.07 (-0.09, -0.05) | 0.15 (0.11, 0.19) |
| 20-30 years | 0.44 (0.39, 0.50) | 0.41 (0.35, 0.48) | -0.04 (-0.06, -0.02) | 0.30 (0.25, 0.35) |
| > 30 years | 0.55 (0.47, 0.62) | 0.49 (0.40, 0.58) | -0.06 (-0.09, -0.03) | 0.39 (0.32, 0.45) |
| *P* for trend | < 0.001 | < 0.001 | < 0.001 | < 0.001 |
| **Model 2** |  |  |  |  |
| 1-10 years | 0 (ref) | 0 (ref) | 0 (ref) | 0 (ref) |
| 10-20 years | 0.09 (0.05, 0.14) | 0.11 (0.05, 0.16) | -0.02 (-0.04, -0.00) | 0.07 (0.02, 0.11) |
| 20-30 years | 0.34 (0.29, 0.40) | 0.27 (0.20, 0.33) | 0.01 (-0.01, 0.03) | 0.21 (0.16, 0.27) |
| > 30 years | 0.43 (0.35, 0.50) | 0.27 (0.19, 0.36) | 0.03 (0.00, 0.06) | 0.27 (0.20, 0.34) |
| *P* for trend | < 0.001 | < 0.001 | 0.006 | < 0.001 |
| **Model 3** |  |  |  |  |
| 1-10 years | 0 (ref) | 0 (ref) | 0 (ref) | 0 (ref) |
| 10-20 years | 0.09 (0.04, 0.14) | 0.09 (0.03, 0.14) | -0.01 (-0.03, 0.00) | 0.06 (0.02, 0.11) |
| 20-30 years | 0.33 (0.27, 0.39) | 0.22 (0.15, 0.28) | 0.02 (0.01, 0.04) | 0.21 (0.16, 0.26) |
| > 30 years | 0.41 (0.34, 0.49) | 0.24 (0.15, 0.32) | 0.04 (0.01, 0.07) | 0.27 (0.20, 0.34) |
| *P* for trend | < 0.001 | < 0.001 | < 0.001 | < 0.001 |

a: Effect estimates (i.e., *slope*) with the 95% *CI* not covering 0 were considered statistically significant.

**Table S5. The effect of ionizing radiation exposure duration on dyslipidemia (categorical index).**

| **Factors** | **Overall dyslipidemia^a^** | **Abnormal TC or TG** | **Hypercholesterolemia** | **Hypertriglyceridemia** | **Hypoalphalipoproteinemia** | **Hyperbetalipoproteinemia** |
| --- | --- | --- | --- | --- | --- | --- |
|  | ***OR* (95% *CI*)** | ***OR* (95% *CI*)** | ***OR* (95% *CI*)** | ***OR* (95% *CI*)** | ***OR* (95% *CI*)** | ***OR* (95% *CI*)** |
| **Model 1** |  |  |  |  |  |  |
| 1-10 years | 1 (ref) | 1 (ref) | 1 (ref) | 1 (ref) | 1 (ref) | 1 (ref) |
| 10-20 years | 1.71 (1.53, 1.92)^b^ | 1.80 (1.59, 2.03) | 1.68 (1.44, 1.96) | 1.78 (1.53, 2.06) | 1.50 (1.25, 1.79) | 1.49 (1.26, 1.76) |
| 20-30 years | 2.25 (1.97, 2.56) | 2.57 (2.24, 2.96) | 2.52 (2.13, 2.98) | 2.29 (1.93, 2.71) | 1.35 (1.08, 1.69) | 2.00 (1.67, 2.41) |
| > 30 years | 3.05 (2.57, 3.61) | 3.41 (2.86, 4.07) | 3.32 (2.71, 4.08) | 2.63 (2.11, 3.26) | 1.51 (1.12, 2.03) | 2.79 (2.23, 3.49) |
| *P* for trend | < 0.001 | < 0.001 | < 0.001 | < 0.001 | < 0.001 | <0.001 |
| **Model 2** |  |  |  |  |  |  |
| 1-10 years | 1 (ref) | 1 (ref) | 1 (ref) | 1 (ref) | 1 (ref) | 1 (ref) |
| 10-20 years | 1.26 (1.11, 1.42) | 1.33 (1.16, 1.51) | 1.36 (1.15, 1.61) | 1.26 (1.07, 1.48) | 1.13 (0.92, 1.37) | 1.21 (1.01, 1.46) |
| 20-30 years | 1.66 (1.44, 1.91) | 1.91 (1.65, 2.22) | 2.05 (1.71, 2.46) | 1.63 (1.36, 1.95) | 1.01 (0.80, 1.28) | 1.65 (1.35, 2.01) |
| > 30 years | 2.05 (1.71, 2.45) | 2.34 (1.94, 2.81) | 2.60 (2.09, 3.23) | 1.65 (1.31, 2.07) | 0.98 (0.72, 1.34) | 2.16 (1.70, 2.74) |
| *P* for trend | < 0.001 | < 0.001 | < 0.001 | < 0.001 | 0.768 | <0.001 |
| **Model 3** |  |  |  |  |  |  |
| 1-10 years | 1 (ref) | 1 (ref) | 1 (ref) | 1 (ref) | 1 (ref) | 1 (ref) |
| 10-20 years | 1.22 (1.08, 1.39) | 1.29 (1.13, 1.48) | 1.34 (1.13, 1.59) | 1.22 (1.03, 1.43) | 1.08 (0.88, 1.32) | 1.20 (1.00, 1.44) |
| 20-30 years | 1.56 (1.35, 1.80) | 1.80 (1.55, 2.10) | 1.99 (1.66, 2.38) | 1.48 (1.23, 1.78) | 0.89 (0.70, 1.14) | 1.61 (1.31, 1.96) |
| > 30 years | 1.98 (1.65, 2.38) | 2.25 (1.87, 2.72) | 2.53 (2.03, 3.15) | 1.54 (1.22, 1.95) | 0.91 (0.67, 1.24) | 2.13 (1.67, 2.70) |
| *P* for trend | < 0.001 | < 0.001 | < 0.001 | 0.012 | 0.172 | <0.001 |

a: Overall dyslipidemia was defined as with at least one abnormal lipid profile.

Abnormal TC or TG: was defined as present either hypercholesterolemia or hypertriglyceridemia, they are the two most common dyslipidemia conditions.

b: Effect estimates (i.e., *OR*) with 95% *CI* not covering 1 were considered.
